# Prenatal diagnosis of fetal syndromes

**DOI:** 10.4103/0971-3026.43845

**Published:** 2008-11

**Authors:** BS Rama Murthy

**Affiliations:** Srinivasa Ultrasound Scanning Centre, 48/2, Shankar Mutt Road, Shankarpuram, Bangalore – 560 004, India

**Keywords:** Fetal, prenatal, syndromes, ultrasound

## Abstract

A syndrome is a pattern of multiple anomalies arising due to a single known causative factor. Ultrasonography has enabled us to recognize many fetal anomalies and dysmorphic features. Recognition of the anomaly pattern leads to the diagnosis of a particular syndrome. This enables us to counsel prospective parents and aids in management. We present a selection of fetal syndromes in the form of a pictorial essay.

A syndrome is a pattern of multiple anomalies thought to be pathogenetically related and not known to represent a single sequence or a polytopic field defect.[1] The constituent anomalies of a syndrome result from a single known cause. The anomalies usually involve multiple systems and do not bear a cause–effect relationship between themselves (i.e., they are not a sequence).

The etiologic factor could be a chromosomal abnormality, a mutant gene defect, an infective agent, or a teratogenic drug or addictive substance. However there are still many situations where the etiology has not been resolved.[2]

Over the past years, the role of the fetal imaging specialist has evolved from just listing the anomalies detected in a fetus to recognizing the specific syndrome or sequence or association of which these anomalies are components of. This facilitates proper counseling of the parents as well as management planning.

The ability to provide as clear information as possible is of great importance during this period of turmoil and confusion in the prospective parents. Such information includes matters related to fetal prognosis, the need for invasive testing, obstetric management plan, short- and long-term neonatal prognosis, and neonatal management decisions. Contact with support groups will go a long way in aiding the parental understanding of the condition. A good resource in this direction is NORD (National Organization for Rare Disorders). Often, it is the imaging/fetal medicine specialist who coordinates the efforts of the obstetrician, geneticist, clinical dysmorphologist, neonatologist, and other relevant specialists.

The suspicion of a set of anomalies/abnormalities being a syndrome may be due to one or more of the following:

1. Known pattern of anomalies: An example is cystic dysplastic kidneys with encephalocele, which arouses the suspicion of Meckel-Gruber syndrome.
2. Recurrence of a set pattern of anomalies: An example is recurrent left diaphragmatic hernia with facial clefting which would suggest the possibility of Fryns syndrome. Recurrence is a hallmark of single-gene defects, which may be autosomal or X linked, recessive or dominant. It becomes very important to get all the information regarding the previous affected pregnancy or pregnancies, including clinical and laboratory details and pictures, if available. An attempt should always be made to examine the affected index child.
3. History of consanguinity.
4. Family history: Construct a pedigree chart.
5. History of teratogenic exposure.

It should be emphasized that a syndrome typically consists of a number of features or anomalies, but a given case may only show a few of these anomalies. In other words, not all cases of the same syndrome would manifest with the same group or combination of anomalies. Again, we can site the example of Meckel-Gruber syndrome. The presence of any two of the following three anomalies, viz, encephalocele, cystic dysplastic kidneys, and postaxial polydactyly, against the background of a normal karyotype would justify the diagnosis of the syndrome. Also, there is the variant of the Meckel-Gruber syndrome which presents with Dandy-Walker malformation instead of an encephalocele.

It is the routine of a structured and detailed fetal USG examination (anomaly scan) that enables the recognition of a syndrome presenting without historical clues. The presence of a syndrome should be especially suspected if multisystem abnormalities are detected. All the anomalies/dysmorphisms detected should be listed and a possible syndromic relationship should be explored. The recognition of a set of anomalies as a potential syndrome is aided by databases such as OMIM (Online Mendelian Inheritance in Man) and LDDB (London Dysmorphology Database).

Whenever there is history of a syndrome in a previous pregnancy or pregnancies, all the features of the syndrome should be noted and those that can be recognized by prenatal USG should be short-listed, and these features or anomalies should be specifically looked for and documented as being present or absent. Many syndromes need detailed scrutiny of the face, extremities, genitalia, etc. Three-dimensional (3D) USG often assists in the study of these anatomical regions. One example is 3D demonstration of midface hypoplasia in craniosynostosis syndromes. A given syndrome has its own natural history and may manifest earlier or later in fetal life or even postnatally. The diagnosis of a syndrome is complete only with a postabortal fetal study (which includes external examination, x-rays, and autopsy) or detailed neonatal examination. Invasive methods like fetal tissue sampling allow a specific diagnosis of chromosomal normality or abnormality. Single-gene syndromes that are suspected on prenatal USG may be confirmed by biochemical, molecular, or cytomolecular techniques. A typical example would be demonstration of unbalanced translocation involving 17p13.3 by fluorescent in situ hybridization in Miller-Dieker syndrome. Some syndromes do not have USG-detectable fetal features. Prenatal molecular diagnosis offers the only means for diagnosis in such instances. Chorion villus sampling, amniocentesis, and fetal blood sampling are the techniques which yield fetal tissue or cells for testing.

Fetal syndromes may be classified on the basis of lethality (or absence of lethality), major system involved, etiology, and so on. It would not be possible here to provide a comprehensive listing and description of all the syndromes. The following are a few examples:

1. Beckwith-Weidemann syndrome (sporadic or AD): The main feature is the triad of macrosomia, omphalocele, and macroglossia. Other features include hepatomegaly, nephromegaly, placentomegaly, and polyhydramnios [Figure 1]. Differential diagnoses to be considered include Down and Zellweger syndromes.

**Figure 1 (a–c):**
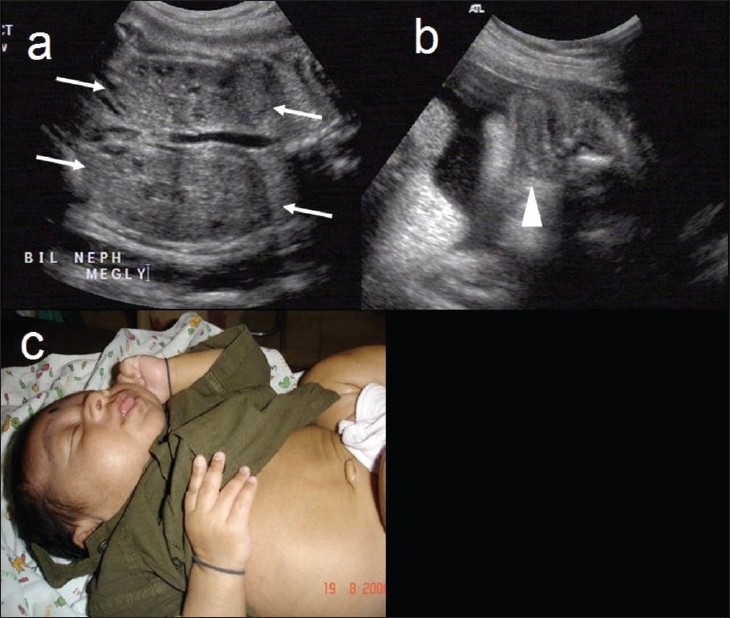
Beckwith-Weideman syndrome. Coronal view of the fetal kidneys (a) shows bilateral nephromegaly (arrows). Coronal view of the fetal face (b) shows macroglossia (arrowhead). Postnatal picture (c) shows macrosomia and macroglossia
2. Walker-Warburg syndrome: (AR) Constituent anomalies are hydrocephalus, agyria (lissencephaly type II), retinal detachment, encephalocele (HARD +/− E syndrome). Other features include cataract, micro-ophthalmia, buphthalmos, and congenital muscular dystrophy [Figure 2].

**Figure 2 (a–d):**
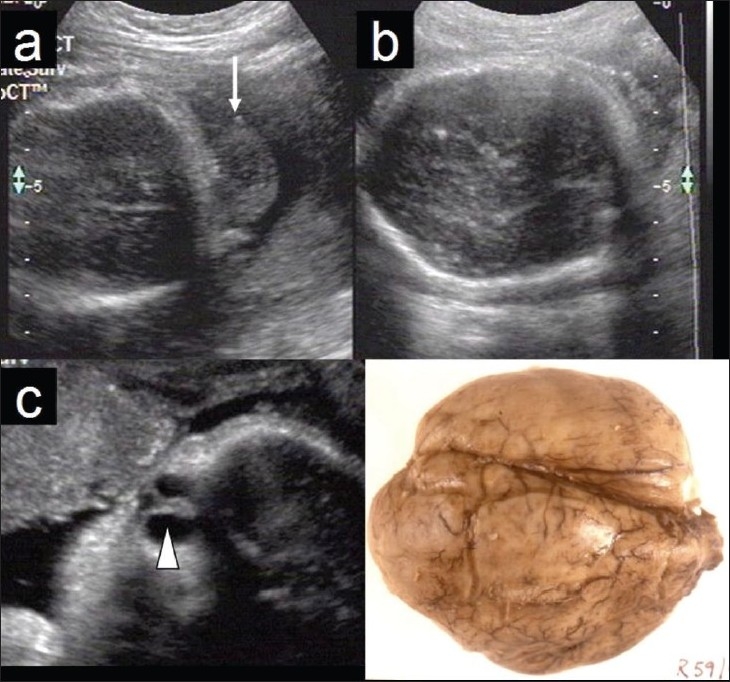
Walker-Warburg syndrome. Axial sections of the fetal cranium show occipital encephalocele (arrow in a) and absent sulci (b). Parasagittal section of the fetal orbit (c) shows retinal detachment (arrowhead). Autopsy picture of the fetal brain (d) demonstrates agyria
3. Wolf-Hirschhorn syndrome: (chromosomal - 4p deletion) Lead clue is severe early-onset IUGR with normal Doppler findings. Other features include ‘Greek helmet’ facial appearance with prominent glabella, rectangular nose, downturned mouth, hypospadias, cardiac and urinary tract defects [Figure 3].

**Figure 3 (a–c):**
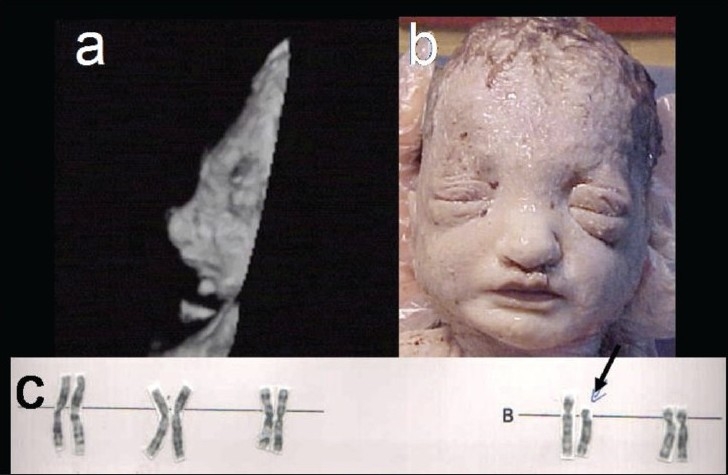
Wolf-Hirschhorn syndrome. Prenatal 3D USG image (a) and postnatal picture of the face (b) show a prominent glabella. Partial karyotype (c) shows 4p deletion (arrow)
4. Turner syndrome: (Chromosomal - monosomy X – 45X0) The main feature is cystic hygroma. Other features include hydrops, IUGR, coarctation of aorta, and horseshoe kidney. Fetal karyotyping clinches the diagnosis [Figure 4]. Differential diagnosis includes Noonan syndrome.

**Figure 4 (a–c):**
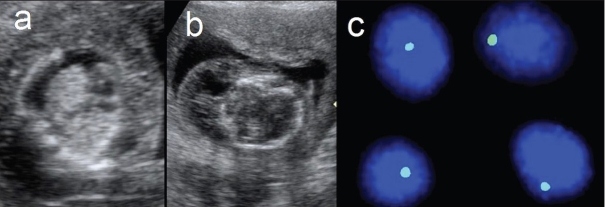
Turner syndrome. Axial section of the fetal thorax (a) shows bilateral pleural effusions. Axial section of the fetal cranium (b) shows a cystic hygroma. Interphase FISH shows a single X chromosome (green fluorescent dot) in each amniocyte
5. Holt-Oram syndrome: (AD) Lead clues are atrial septal defect, ventricular septal defect or atrioventricular septal defect, and radial ray defect (absence of thumb). Differential diagnosis includes thrombocytopenia absent radius syndrome, Fanconi syndrome, and VACTERL association [Figure 5].

**Figure 5 (a–c):**
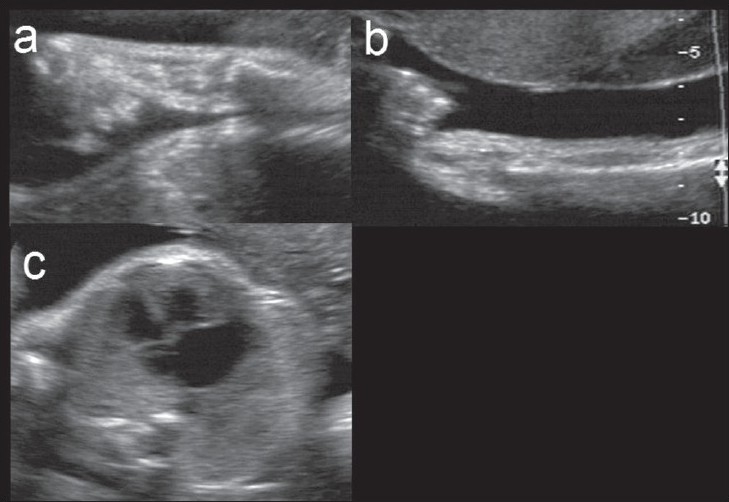
Holt-Oram syndrome. Fetal right (a) and left (b) forearms show radial aplasia and club hand. Four-chamber view of the heart (c) shows an atrioventricular septal defect
6. Meckel-Gruber syndrome: (AR) The triad of cystic dysplastic kidneys, occipital encephalocele, and post-axial polydactyly typifies this syndrome. Other features include posterior cranial fossa abnormalities and facial clefting. The cystic dysplastic kidneys (enlarged echogenic kidneys) are always present in this syndrome and and results in oligohydramnios or anhydramnios [Figure 6].

**Figure 6 (a–c):**
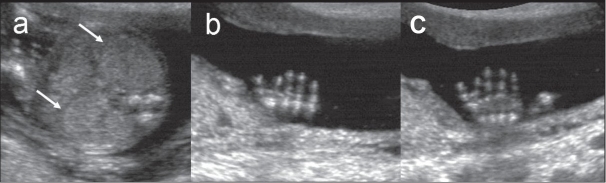
Meckel-Gruber syndrome. Axial section of the abdomen (a) shows bilateral enlarged hyperechoic kidneys (arrows). Sections through right and left hands (b and c respectively) show bilateral postaxial polydactyly
7. Crouzon syndrome: (AD) Lead clues are craniosynostosis, frontal bossing, proptosis, and hypertelorism. Other features include beaked nose and cloverleaf skull. Differential diagnoses will include other craniosynostosis syndromes like Pfeiffer, Apert, Carpenter, or Sathre-Chotzen syndromes [Figure 7].

**Figure 7 (a–e):**
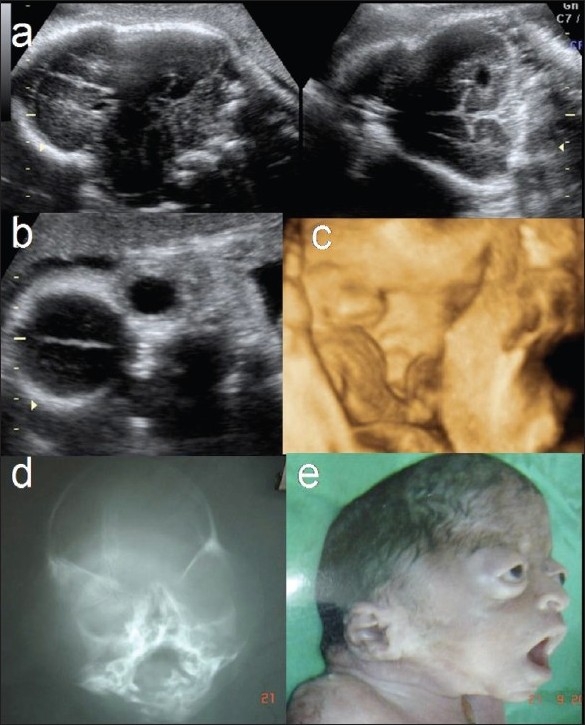
Crouzon syndrome. Axial and coronal sections of the fetal cranium (a) show a fused coronal suture and a clover-leaf calvarium. Coronal section of the face (B) shows prominent orbits. Surface rendering of fetal face (c) shows proptosis. Anteroposterior radiograph of the skull (d) and postabortal picture (e) of the abortus show typical features
8. Klippel-Trenaunay-Weber syndrome: (sporadic) This syndrome typically features multiple soft-tissue hemangiomas in the subcutaneous plane. Other features include limb hypertrophy, and edema [Figure 8]. Differential diagnosis includes Proteus syndrome.

**Figure 8 (a–d):**
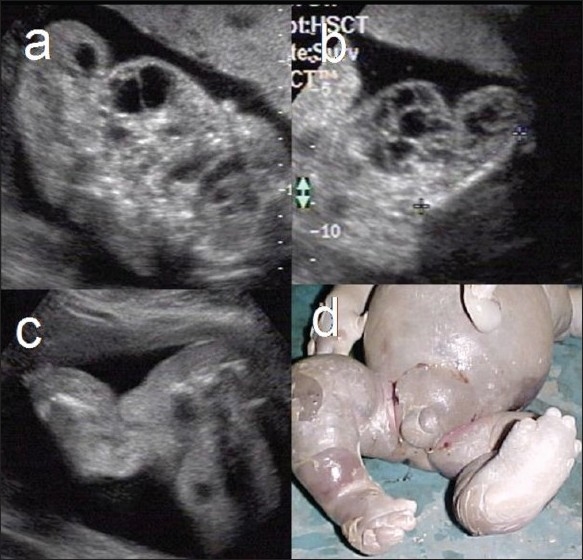
Klippel-Trenaunay-Weber syndrome. Subcutaneous, septate, cystic space-occupying lesions are seen in the right thigh (a) and left arm (b) regions, with extensive subcutaneous edema (c) in the left leg. Postabortal picture (d) of the trunk and lower limbs shows extensive edema
9. Pseudo-TORCH syndrome: (AR) Lead clue is cerebral atrophy with periventricular and basal ganglia calcification. Other features include microcephaly, hepatomegaly, splenomegaly, and thrombocytopenia [Figure 9]. Cordocentesis to prove absence of fetal infection and presence of thrombocytopenia is indicated. Fetal MRI would be of help to confirm cerebral atrophy. Differential diagnoses of Aicardi-Goutiere's syndrome and TORCH infection may be considered.

**Figure 9 (a–e):**
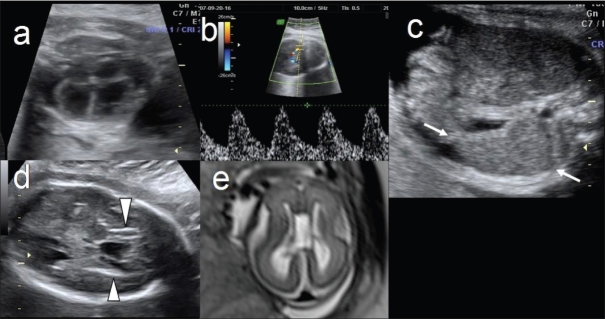
Pseudo-TORCH syndrome. Fetal cardiomegaly (a), elevated middle cerebral artery velocity (greater than 1.5 MoM) (b), fetal splenomegaly (arrows in c) and fetal periventricular calcification (arrowheads in d) are seen. Fetal cranial MRI (e) shows cerebral atrophy. All these features coupled with a negative maternal serology for infection, are diagnostic
10. Fetal toxoplasmosis syndrome: (transplacental infection by a protozoan, *Toxoplasma gondii*) Typical features of this syndrome would include hydrocephalus and cerebral calcification. Other features include microcephaly, hepatosplenomegaly, and ascites [Figure 10]. Confirmation is by PCR detection of the infective agent in amniotic fluid.

**Figure 10:**
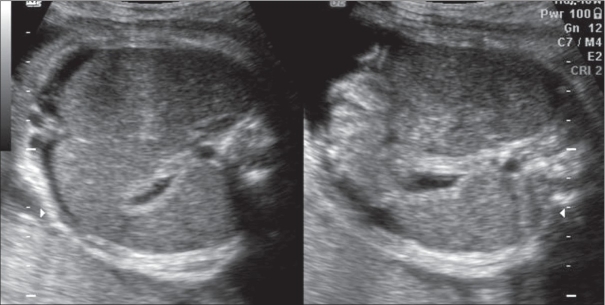
Toxoplasmosis. Fetal hepatosplenomegaly is seen with ascites. Maternal serology was positive for toxoplasma

Thus we see that prenatal USG is capable of recognizing syndromic patterns of anomalies. This is of great importance and has direct management implications.
